# The impact of mid-wall striate of LGE at interventricular septum to the beta-broker (Carvedilol) titrating to the target dose and the improvement of cardiac function with DCM

**DOI:** 10.1186/1532-429X-13-S1-P274

**Published:** 2011-02-02

**Authors:** Kunihiko Teraoka, Masafumi Kawade, Yoshinori Suzuki, Kenji Takazawa, Akira Yamashina

**Affiliations:** 1 Tokyo Med.University, Tokyo, Japan

## Introduction

It was reported that mid-wall striate late gadolinium enhancement at interventricular septum (MWS-LGE) definite by CMR was strong predictor of prognosis including sudden death with dilated cardiomyopathy (DCM). On the other hand, randomized trial have shown that beta-brokers lead symptomatic improvement, reduced hospitalization and enhanced survival in many patients with heart failure.

## Purpose

The object of this study was to evaluate the impact of mid-wall striate of LGE(MWS-LGE) at interventricular septum to beta-broker (Carvedilol) titrating to the target dose and improvement of cardiac function with DCM.

## Methods

Fifty-five patients with DCM examined by LGE-CMR were enrolled. They were treated with Carvedilol at higher dose as possible.

They were divided into two groups according to MWS-LGE positive or not.

The maximum dose of Carvedilol with each group at chronic stable condition(after 21.6 +/- 21.4 months later from initiation of Carvedilol) were compared. The improvement of UCG parameter of each group by Carvedilol therapy was examined.

## Results

1) The UCG parameters of all 55 DCM patients recorded just before initiation of Carvedilol were LVDd; 65.2±7.5mm,LVDs; 56.3±8.5mm,EF;28.2.±8.1% and mean final dose of Carvedilol with all 55 DCM patients was 11.5±5.5mg.

2) MWS-LGE was found in twenty patients of DCM (38.1%) with LGE-CMR.

3) There were no significant difference between two groups in LVDd, LVDs and EF(P>0.79,P>0.92,P>0.76, respectively)at first echocardiograph.

4) The final dose of carvedilol with MWS-LGE -positive group was lower than with MWS-LGE -negative group (9.2+/-5.4 mg VS 13.0 +/-5.1mg, respectively).

5) The UCG parameter showed improvement at chronic stable phase compared to those had recorded at just before initiation of Carvedilol in the both groups, but especially in the MWS-LGE negative group showed a greater improvement than those of MWS-LGE positive group (Table 1).

**Table 1:** The UCG parameters at chronic stable phase

MWS-LGE	LVDd(mm)	LVDs(mm)	EF(%)
Negative	54.4+/-7.7	39.8+/-9.9	52.3+/-14.2
Positive	63.1+/-10.9	52.4+/-14.8	36.2+/-18.1
P-value	<0.02	<0.02	<0.01

## Conclusions

The relationship between the beneficial effect of Carvedilol with DCM and MWS-LGE defined by LGE-MRI was examined. The DCM patients with MWS-LGE negative showed higher maximum dose of Carvedilol and greater improvement of UCG parameters compared to those of patients with MWS-LGE positive. The MWS-LGE has a potential to have a strong worse impact to titrating and beneficial effect of Carvedilol with DCM.

